# Median Arcuate Ligament Syndrome in 17-year-old Male with Abdominal Pain: Case Report

**DOI:** 10.5811/cpcem.1510

**Published:** 2024-01-23

**Authors:** Jessica Doctor, Jonathan Henderson

**Affiliations:** San Antonio Uniformed Services Health Education Consortium, Fort Sam Houston, TX

**Keywords:** *median arcuate ligament syndrome*, *angiogram*, *case report*

## Abstract

**Introduction:**

Median arcuate ligament syndrome (MALS) is an uncommon cause of chronic abdominal pain resulting from the compression of the celiac artery. It shares symptoms with chronic functional abdominal pain, a more common cause of pediatric chronic abdominal pain. Typically found in middle-aged females, MALS is a diagnosis of exclusion.

**Case Report:**

A 17-year-old male who presented to the emergency department with periumbilical pain for two months was subsequently diagnosed with MALS through computed tomography angiography. Further vascular and gastroenterology evaluations confirmed the diagnosis, ruling out other common causes of chronic abdominal pain. The patient received non-operative treatment in the form of endoscopic ultrasound celiac plexus block, with the possibility of surgical management if necessary.

**Conclusion:**

Median arcuate ligament syndrome is an uncommon cause of chronic abdominal pain that is difficult to differentiate from other causes, especially in pediatric patients. It should be considered in the patient whose previous workup was not conclusive and symptom management had failed. Management is multidisciplinary with non-operative management preferred initially. If there is no improvement, surgical management should be considered.

Population Health Research Capsule What do we already know about this clinical entity?

*Median arcuate ligament syndrome (MALS) is a rare cause of chronic abdominal pain due to celiac artery compression, typically diagnosed in females 30–50 years old.*

What makes this presentation of disease reportable?

*A male pediatric patient was diagnosed with MALS via computed tomography angiogram rather than the usual mesenteric duplex ultrasound.*

What is the major learning point?

*Vascular pathology should be considered as a cause of chronic abdominal pain in pediatric patients whose clinical picture does not fit other common diagnoses.*

How might this improve emergency medicine practice?

*This case report may broaden an emergency physician’s differential diagnosis and change workup by ordering the appropriate study.*

## INTRODUCTION

Median arcuate ligament syndrome (MALS), also known as celiac artery compression syndrome or Dunbar syndrome, is characterized by the compression of the celiac artery by the median arcuate ligament—a fibrous arch connecting the diaphragmatic crura. This rare condition presents with symptoms of foregut ischemia, including postprandial or exercise-induced abdominal pain, nausea, vomiting, and weight loss.^1^ These symptoms often overlap with those of chronic functional abdominal pain (CFAP), commonly attributed to conditions such as irritable bowel syndrome (IBS).^2^ The etiology and pathophysiology of MALS remain poorly understood but are believed to involve both ischemic and neuropathic components. While perfusion to the intestines is usually unaffected due to collateral circulation, the proximity of the celiac ganglion to the compressed celiac artery and the observed symptomatic improvement following interventions targeting the celiac ganglion suggest a neuropathic contribution. Median arcuate ligament syndrome typically presents more commonly in females (4∶1 ratio) and is more common between the ages of 30–50 years in those with a thin habitus. It can occur in pediatric patients.^1^

## CASE REPORT

A 17-year-old, otherwise healthy White male was brought in by his mother to the emergency department (ED) for abdominal pain that had been constant for two months. He was previously seen at an urgent care and unsuccessfully treated for a urinary tract infection despite having normal urinalysis. He had followed up with his pediatrician and was referred to the ED for further computed tomography (CT) imaging and laboratory studies. He described his pain as sharp, located in the umbilical region, and worse with physical activity. Pain was not worse with eating. He’d had intermittent nausea and vomiting since he was a child. The patient was notably thin and had a lifetime of short stature and being underweight, but his growth curve was otherwise tracking. He typically had one to two bowel movements daily without any pain relief.

Due to concerns for a vascular etiology of his abdominal pain, a CT angiogram of the abdomen and pelvis was obtained in the ED, which demonstrated narrowing of the origin of the celiac artery at the location of median arcuate ligament and post stenotic dilation (Image). The celiac artery was otherwise patent. The rest of his CT showed normal findings. His other workup in the ED, including a complete blood count with differential, comprehensive metabolic panel, and lipase, was unremarkable. The patient subsequently followed up with the vascular surgery outpatient clinic, where vascular ultrasound demonstrated significant velocity elevations in the celiac artery at rest, further increasing with end expiration—an indicative finding of MALS. He was referred to gastroenterology for further evaluation of other, more common causes of abdominal pain.

**Image.**
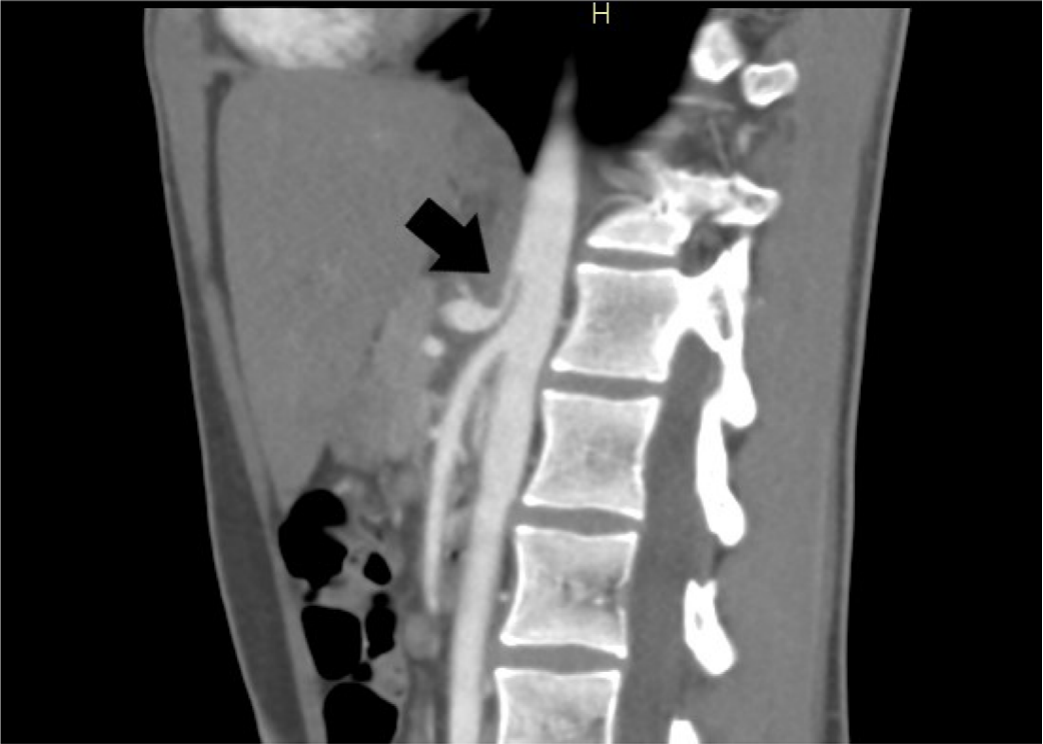
Computed tomography angiography abdomen and pelvis demonstrating narrowing of the celiac artery in a J-shaped configuration with post stenotic dilation seen in median arcuate ligament syndrome (arrow).

During subsequent months, the patient underwent a comprehensive gastroenterological workup, which included negative celiac antibodies, normal erythrocyte sedimentation rate, normal C-reactive protein, and esophagogastroduodenoscopy. The endoscopy revealed normal findings in the esophagus, stomach, and duodenum, with biopsies indicating minimal evidence of acid reflux but did not otherwise explain his periumbilical pain. The patient trialed esomeprazole for acid reflux with minimal improvement observed. The gastroenterology team suggested trying sertraline or amitriptyline for neuropathic gastrointestinal pain, but the family declined this option. Instead, they opted for conservative management of MALS, focusing on non-operative treatment through endoscopic ultrasound celiac plexus block. Post-treatment follow-up will determine whether surgical management is necessary in the absence of significant symptom improvement.

## DISCUSSION

Median arcuate ligament syndrome is an infrequent cause of abdominal pain, which frequently presents as a primary complaint in ED visits. It is less commonly diagnosed in pediatric males and often mimics CFAP symptoms, leading to misdiagnoses such as IBS, functional dyspepsia, or abdominal migraine.^2^ This case highlights the importance of considering vascular etiologies such as MALS in pediatric patients whose clinical presentation does not align with other common causes of chronic abdominal pain. The patient’s repeated visits and investigations underscore the challenges in diagnosing this condition, which is typically confirmed using mesenteric duplex ultrasound as a non-invasive first-line option, or CT angiography.^1^

The management of MALS varies due to the multifaceted nature of its etiology. Treatment strategies aim to alleviate the vascular or neuropathic sources of pain. In this case, the patient and his family decided to pursue non-surgical management through celiac plexus block. Other non-operative approaches include a multidisciplinary approach involving general surgery, vascular surgery, and psychiatry. The involvement of psychiatry is crucial not only for managing the stress associated with potential surgery but also for addressing concurrent psychiatric conditions, such as depression and eating disorders, commonly observed in these patients. Surgical management typically entails the release of the median arcuate ligament through laparoscopic, robotic, or open procedures, all of which have been proven to be safe and effective.^3^ ^,^ ^4^ However, it should be noted that prolonged compression of the celiac artery may induce structural changes in the vascular layers, including intimal hyperplasia, media elastin proliferation, and adventitial disorganization. These changes may explain why surgical release of the median arcuate ligament does not consistently alleviate symptoms.^5^

## CONCLUSION

Median arcuate ligament syndrome is a rare cause of chronic abdominal pain that poses a diagnostic challenge, particularly in pediatric patients. Vascular etiologies such as MALS should be considered when previous workup and management have failed to provide a definitive diagnosis. A multidisciplinary approach to management is essential, with non-operative treatment recommended initially. Surgical management, involving the release of the median arcuate ligament, should be considered if conservative measures fail to yield significant symptom improvement.

## References

[1] Goodall R Langridge B Onida S et al. Median arcuate ligament syndrome. J Vasc Surg. 2020;71(6):2170–6. 31882314 10.1016/j.jvs.2019.11.012

[2] Mak GZ Speaker C Anderson K et al. Median arcuate ligament syndrome in the pediatric population. J Pediatr Surg. 2013;48(11):2261–70. 24210197 10.1016/j.jpedsurg.2013.03.003 PMC3896126

[3] Skelly CL Mak GZ. Median arcuate ligament syndrome: current state of management. Semin Pediatr Surg. 2021;30(6):151129. 34930594 10.1016/j.sempedsurg.2021.151129

[4] Duffy AJ Panait L Eisenberg D et al. Management of median arcuate ligament syndrome: a new paradigm. Ann Vasc Surg. 2009;23(6):778–84. 19128929 10.1016/j.avsg.2008.11.005

[5] Bech F Loesberg A Rosenblum J et al. Median arcuate ligament compression syndrome in monozygotic twins. J Vasc Surg. 1994;19(5):934–8. 8170050 10.1016/s0741-5214(94)70021-4

